# Diagnosing Schistosomiasis by Detection of Cell-Free Parasite DNA in Human Plasma

**DOI:** 10.1371/journal.pntd.0000422

**Published:** 2009-04-21

**Authors:** Dominic Wichmann, Marcus Panning, Thomas Quack, Stefanie Kramme, Gerd-Dieter Burchard, Christoph Grevelding, Christian Drosten

**Affiliations:** 1 Sektion Infektiologie und Tropenmedizin, I. Medizinische Klinik, Universitätsklinikum Eppendorf, Hamburg, Germany; 2 Clinical Virology Group, Bernhard Nocht Institute for Tropical Medicine, Hamburg, Germany; 3 Institute of Parasitology, Faculty of Veterinary Medicine, Justus Liebig University, Giessen, Germany; René Rachou Research Center, Brazil

## Abstract

**Introduction:**

Schistosomiasis (bilharzia), one of the most relevant parasitoses of humans, is confirmed by microscopic detection of eggs in stool, urine, or organ biopsies. The sensitivity of these procedures is variable due to fluctuation of egg shedding. Serological tests on the other hand do not distinguish between active and past disease. In patients with acute disease (Katayama syndrome), both serology and direct detection may produce false negative results. To overcome these obstacles, we developed a novel diagnostic strategy, following the rationale that *Schistosoma* DNA may be liberated as a result of parasite turnover and reach the blood. Cell-free parasite DNA (CFPD) was detected in plasma by PCR.

**Methodology/Principal Findings:**

Real-time PCR with internal control was developed and optimized for detection of CFPD in human plasma. Distribution was studied in a mouse model for *Schistosoma* replication and elimination, as well as in human patients seen before and after treatment. CFPD was detectable in mouse plasma, and its concentration correlated with the course of anti-*Schistosoma* treatment. Humans with chronic disease and eggs in stool or urine (n = 14) showed a 100% rate of CFPD detection. CFPD was also detected in all (n = 8) patients with Katayama syndrome. Patients in whom no viable eggs could be detected and who had been treated for schistomiasis in the past (n = 30) showed lower detection rates (33.3%) and significantly lower CFPD concentrations. The duration from treatment to total elimination of CFPD from plasma was projected to exceed one year.

**Conclusions/Significance:**

PCR for detection of CFPD in human plasma may provide a new laboratory tool for diagnosing schistosomiasis in all phases of clinical disease, including the capacity to rule out Katayama syndrome and active disease. Further studies are needed to confirm the clinical usefulness of CFPD quantification in therapy monitoring.

## Author Summary

Bilharzia (schistosomiasis) occurs in the tropics and subtropics and is one of the most important parasite diseases of humans. It is caused by flukes residing in the vessels of the gut or bladder, causing fever, pain, and bleeding. Bladder cancer or esophageal varices may follow. Diagnosis is difficult, requiring detection of parasite eggs in stool, urine, or gut/bladder biopsies. In this paper, we introduce a fundamentally new way of diagnosing bilharzia from the blood. It has been known for almost 20 years that patients with cancer have tumor-derived DNA circulating in their blood, which can be used for diagnostic purposes. During pregnancy, free DNA from the fetus can be detected in motherly blood, which can be used for diagnosing a range of fetal diseases and pregnancy-associated complications. We found that parasite DNA can be detected in the same way in the blood of patients with bilharzia. In patients with early disease, diagnosis was possible earlier than with any other test. DNA could be detected in all patients with active disease in our study. Patients after treatment had significantly lower parasite DNA concentrations and turned negative 1–2 years after treatment. Future studies should implement the method in large cohorts of patients and should define criteria for the confirmation of the success of treatment by comparing the concentration of fluke DNA before and after therapy.

## Introduction

Schistosomiasis, also known as bilharzia, is caused by trematodes of the *Schistosomatidae* family. It is among the most important parasitic diseases worldwide, with a significant socio-economic impact [1]. More than 200 million people are infected, and about 200,000 may die from the disease each year. On a global scale, one of thirty individuals has schistosomiasis [2]. Movements of refugees, displacement of people, and mistakes in freshwater management promote the spread of schistosomiasis [3],[4].

Human disease is caused by *S. haematobium*, *S. mansoni*, *S. japonicum*, and less frequently, *S. mekongi* and *S. intercalatum* [5],[6]. Infection with *cercariae* occurs through intact skin via contact with infested water. Penetration of *cercariae* is followed by Katayama syndrome, an acute syndrome with fever, rash and eosinophilia. The syndrome is thought to be caused by antigen excess due to the presence of schistosomules in blood and the beginning of egg deposition [5],[6]. After maturation in the lung and liver sinusoids, adult male and female worms mate and actively migrate to their target organs [4],[5]. *S. haematobium* resides in walls of the bladder and sacral and pelvic blood vessels surrounding the urinary tract. The other mentioned species reside in mesenteric veins. After deposition of eggs in the capillary system, eggs penetrate the mucosa of target organs and are excreted in urine or feces. Sequelae of acute and chronic infection include hepato-splenic disease, portal hypertension with varices, pulmonary hypertension, squamous cell cancer of the bladder, liver fibrosis, and less common conditions such as myelo-radiculitis and female genital schistosomiasis. Co-infections with HCV and *Schistosoma* may also modify the course of hepatitis C [4], [7]–[14].

Anti-*Schistosoma* antibodies can be detected by enzyme immunoassay (EIA), immunofluorescence assay (IFA), and indirect hemagglutination assay [15]. Antibody detection is valuable in patients with rare exposure to *Schistosoma*, e.g., tourists. In patients with Katayama syndrome, a positive EIA antibody test is usually the earliest diagnostic laboratory result. Still, a large fraction of patients will initially test negative [16],[17]. False negative tests prevent timely treatment of schistosomiasis in travellers who present with fever of unknown origin. Moreover, the inability of serology to discriminate between active and past disease limits its clinical value for confirmation of the success of treatment [18].

Microscopic demonstration of eggs in stool or urine specimens is considered the diagnostic gold standard for confirmation of schistosomiasis in patients from endemic countries, as well as for the confirmation of the success of treatment. In field studies the rapid and inexpensive Katz-Kato thick smear technique is often used [19]. Because the shedding of eggs is highly variable, it is necessary to concentrate eggs from stool or urine prior to examination [20]. Even in concentrated samples the sample volume analysed in the microscope is limited. Due to random distribution effects, the analysed sample may not contain eggs even if the disease is active. It is thus very difficult to achieve a conclusive confirmation of successful therapy. In symptomatic patients with unsuccessful egg detection, it is often necessary to perform endoscopic biopsies of the bladder or rectal mucosa to increase the chance of detection [20].

Several groups have developed polymerase chain reaction (PCR) methods to improve the direct detection of *Schistosoma*. These tests are done on urine, stool, or organ biopsy samples, and involve the preparation of DNA from eggs prior to PCR amplification [21],[22]. Unfortunately, only a small volume of sample can be processed for DNA extraction, and it is dependent on chance whether the processed sample contains eggs or not. In this regard, PCR has the same limitations as microscopy and does not provide a significant clinical benefit.

The detection of circulating cell-free DNA in human plasma has long been explored for the non-invasive diagnosis of a variety of clinical conditions (reviewed in [23] and [24]). It has been known since almost 20 years that patients with solid tumors have tumor-derived DNA circulating in plasma that can be used for diagnostic purposes [25]–[28]. Circulating fetal DNA in maternal plasma is used for diagnosing and monitoring of a range of fetal diseases and pregnancy-associated complications [29]–[33]. The normal concentration of cell-free DNA in plasma of adults is 10–100 ng/mL or 10e3 to 10e4 human genome equivalents per mL [34],[35]. It has been determined that the concentration of fetal DNA in maternal plasma is 3.4% of total serum DNA on average [16]. The presence of cell-free DNA in plasma may be a consequence of apoptosis, which is associated with physiological and pathological turnover of tissue, e.g., in tumor growth or embryonic development (reviewed in [36] and references therein).

In parasitic diseases such as schistosomiasis, there is a huge turnover of parasites involving replication, maturation, and death of organisms. Multi-cellular parasites like *Schistosoma* contain DNA copies in stoichiometrical excess over parasite count. We reasoned that it might be possible to find cell-free parasite DNA (CFPD) circulating in plasma, and that this could be used to diagnose schistosomiasis. In contrast to eggs in stool or urine, CFPD would be equally distributed throughout the plasma volume of the patient, resolving the issue of random sampling that spoils clinical sensitivity of classical detection methods. As an extension of this rationale, we reasoned that it might also be possible to confirm the elimination of *Schistosoma* CFPD after successful treatment. To prove these concepts, *Schistosoma*-specific real-time PCR was established and optimised for detection of DNA from large volumes of plasma. A Balb/c mouse model of schistosomiasis was used to study the levels of CFPD in plasma during infection, as well as during and after therapy. The concept was then transferred to patients with different stages of infection, including Katayama syndrome, chronic disease with egg excretion, and patients treated for schistosomiasis in the past without current signs of disease.

## Materials and Methods

### Ethics statement

Written informed consent was obtained from every patient. The study was approved by the ethics committee of the Board of Physicians of the City of Hamburg.

### Animal model of *S. mansoni* infection

The Liberian isolate of *S. mansoni* [37], was maintained in *Biomphalaria glabrata* and Syrian hamsters. Maintenance of the life cycle was exactly performed as described elsewhere [38]. Adult female Balb/c mice (Charles Rivers Laboratories, Sulzfelden, Germany) were infected by intraperitoneal injection of 100 cercariae diluted in 200 µL sterile isotonic saline solution. Approval was obtained from the animal protection board of the City of Hamburg.

### Patients

The study included patients with Katayama syndrome (n = 8) defined by fever, eosinophilia and a history of surface freshwater contact during a recent travel to a schistosomiasis endemic region. A second group had active, untreated disease defined by detection of eggs in stool or urine (n = 14). Most patients in this group were immigrants from endemic regions presenting to their primary care physician with acute manifestations like hematuria. Most of them where not aware of their disease. A third group of patients had treated schistosomiasis defined by prior anti-parasitic treatment and failure to detect viable eggs by microscopy (n = 30).

### Samples

Serum and plasma samples were collected for antibody testing and DNA extraction, respectively. Serum was stored at +4°C and plasma was stored at −20°C prior to use. Stool samples were collected in Merthiolat-Iodine-Formol buffer and stored at +4°C until use.

### Serology

All patient sera were tested for anti-*Schistosoma* antibodies by means of an extensively validated in-house EIA that has been described previously [15]. EIA used crude extracts from cercariae and adult worms of *S. haematobium* and *S. mansoni*, as well as extracts from adult worms of *S. japonicum*.

### Parasite detection

Stool investigation was done essentially as described earlier [15]. For detection of *S. haematobium* eggs, urine was filtered as described by Peters et al. [39]. Microscopy was performed directly on untreated biopsies and on paraffin-embedded tissue. The latter was cut with a microtome into 5-µm sections. The sections were subsequently mounted on glass slides, stained with hematoxylin-eosin, periodic acid–Schiff and Trichrome stains and subsequently examined by an experienced pathologist for *Schistosoma* eggs.

### Plasma DNA preparation

DNA from plasma was prepared by large volume phenol-chloroform extraction. In brief, up to 20 mL of plasma were mixed with an equal volume of phenol and centrifuged for 5 min at 1,200 g. The aqueous phase was transferred to a new tube, mixed with an equal volume of phenol∶chloroform 1∶1, and centrifuged 5 min at 3,500 rpm. Again the aqueous phase was transferred to a new tube, mixed with an equal volume of chloroform, and centrifuged 5 min at 3,500 rpm. DNA was precipitated by adding 1/10 volume of 3 M sodium acetate and 1 volume of 99% ethanol. After centrifugation for 1 h at 14,000 g the supernatant was discarded. To remove residual salt the pellet was washed with 1 mL ethanol 70% and centrifuged 20 min at 10,000 rpm. Supernatant was discharged. The DNA pellet was air-dried and dissolved in 50 µL of water and stored at −20°C.

### Real-time PCR

In order to achieve high analytical sensitivity, the 121 bp tandem repeat sequence (GenBank accession number M61098) that contributes about 12% of the total *Schistosoma mansoni* genome sequence was chosen as the PCR target gene [40]. 20 µL reactions contained 3 µL of DNA, 2 µL 10X Platinum Taq PCR-Buffer (Invitrogen, Karlsruhe, Germany), 1.5 µL MgCl_2_ (50 µM), 200 µM of each dNTP, 0.8 µg bovine serum albumin, 500 nM of primers SRA1 (CCACGCTCTCGCAAATAATCT and SRS2 (CAACCGTTCTATGAAAATCGTTGT) each, 300 nM of probe SRP (FAM-TCCGAAACCACTGGACGGATTTTTATGAT-TAMRA), and 1.25 units of Platinum Taq polymerase (Invitrogen). Cycling in a Roche LightCycler® version 1.2 comprised: 95°C/5 min, 45 cycles of 58°C/30 s and 95°C/10 s. Fluorescence was measured once per cycle at the end of the 58°C segment.

### Quantification standard and technical sensitivity

The PCR target fragment was cloned into plasmid by means of a pCR 2.1-TOPO TA cloning reagent set (Invitrogen, Carlsbad, California, USA). Plasmid purification was done with a QIAprep MiniPrep kit (Qiagen, Hilden, Germany). Plasmids were quantified by spectrophotometry. The standard plasmid was tested in 10-fold dilution series by PCR, showing a detection limit of 5.4 copies per reaction. If plasmids were inoculated into 200 µL of plasma prior to preparation, 68.8 copies per mL of plasma were detectable. Because the DNA contained in 200 µL was concentrated in 50 µL elution volume, of which 3 µL were tested by PCR, the PCR input at 68.8 copies per mL corresponded to a calculated 0.8 copies per PCR. Dilutions of the standard plasmid were also used as a quantification reference in real-time PCR. It should be mentioned that only approximate concentrations of *Schistosoma* DNA can be determined because the number of copies per genome of our target sequence varies between *S.mansoni* and *hematobium* and is unknown for *S. japonicum* [40].

### Internal control

Target gene nucleotides 39–79 bp were removed from the quantification standard plasmid, and replaced by an alternative probe binding site with techniques described earlier [41], using primers SRA-mut (ATCGTTCGTTGAGCGATTAGCAGTTTGTTT TAGATTATTTGCGGAGCGTGG) and SRS2-mut (CTGCTAATCGCTCAACGAAC GATTACAACGATTTTCATAGAACGGTTGG) for extension PCR, in combination with diagnostic PCR primers mentioned above. The resulting construct was cloned as described in the section “quantification standard”.

### Optimization of *Schistosoma* PCR for large plasma volumes

One whole schistosome was ground in liquid nitrogen. Its nucleic acids were extracted and inoculated into human normal plasma. Different volumes of plasma were prepared by classical phenol-chloroform extraction, keeping the water volume in which DNA was resuspended at the end of the procedure constant at 50 µL. Parallel PCRs conducted on these nucleic acid solutions showed that an increase of detection signal was achieved up to an input volume of 10 mL of plasma, as evident by reduction of Ct values in real-time PCR. Above this input volume, no increase of signal was observed anymore, probably due to the introduction of interfering substances into PCR that were derived from large volumes of plasma. These experiments were repeated and confirmed with plasmid DNA spiked in human plasma. An input volume in humans of 10 mL of plasma was chosen as the volume to be analyzed in human diagnostic application in this study. It should be mentioned that outside this study, smaller volumes of plasma (down to 1 mL) were successfully used for CFPD detection.

### Quantitative correction factors

Different input volumes of plasma were processed for mice or humans, respectively. For mice, 1 mL of plasma was extracted and the resulting DNA resuspended in 50 µL, of which 3 µL were tested in PCR. One DNA copy per PCR vial thus represented 16.7 copies per mL (50 / 3). For Humans, 10 mL of plasma were extracted and resuspended in 50 µL of water, of which 3 µL were tested in PCR. One DNA copy per PCR vial thus represented 1.67 copies per mL.

### Confirmation of PCR specificity

Plasma from 30 blood donors and 35 patients examined for other conditions were tested by large-volume plasma extraction and CFPD real-time PCR. None yielded positive results.

### Statistical analysis

The Statgraphics V 5.1 software package (Manugistics, Dresden, Germany) was use for all statistical analyses. T-tests were always two-tailed.

## Results

In the case of tumors and pregnancy, cell-free DNA can be detected in plasma. Because the high turn-over rates of cells in these conditions resemble processes observed in parasitic infections, we reasoned that the detection of cell-free DNA from infecting parasites (CFPD) might be effective as a diagnostic approach in schistosomiasis. In preliminary experiments, stored serum samples from humans with confirmed schistosomiasis were processed with a method commonly used for detection of DNA viruses from cell-free plasma [42] and tested by *Schistosoma* PCR [21]. Plasma samples from mice infected with *S. mansoni* were also tested. In both cases, *Schistosoma* DNA was detectable in some but not all of the tested samples (data not shown).

**Figure 1:**
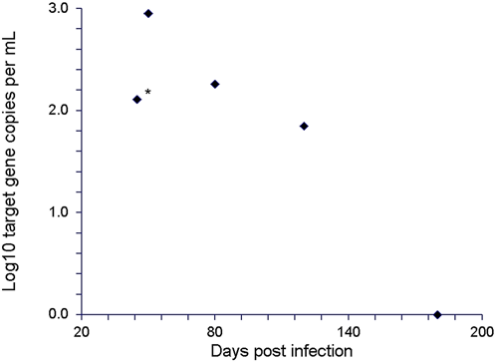
DNA copies per mL of pooled mouse plasma (y-axis, four mice per datum point) in mice infected intraperitoneally with 100 cercariae of *S. mansoni*. After completion of parasite maturation on day 42, mice were treated orally with praziquantel on day 45 (120 mg per kg). At the indicated times (x-axis), four mice were sacrificed, their blood pooled, and 1 mL of pooled plasma was tested as described in the Materials and Methods section for cell-free *Schistosoma* DNA. The untreated group is marked with an asterisk (*).

To determine systematically under which conditions and at what quantities CFPD was detectable in schistosomiasis, a quantitative real-time PCR assay for a *Schistosoma* multi-copy gene was established as described in the Materials and Methods section. A well-established mouse model of schistosomiasis was employed. In a first step it was tested whether CFPD circulated in plasma during the phase of chronic schistosomiasis. Four adult BALB/c mice were infected with 100 cercariae of *S. mansoni* and sacrificed after completion of the replication cycle on day 42 after infection. To enable testing of a large volume of mouse plasma, blood was pooled from four mice and one mL of pooled plasma was extracted. Quantitiative PCR with an absolute quantification standard (refer to Materials and Methods section) yielded a DNA concentration of 128.27 copies of CFPD target gene per mL of plasma (Figure 1, marked datum point).

It was next determined whether any associations might exist between the amount of living parasites in mice and the concentration of CFPD. Along with the four mice mentioned above, 16 more mice had been infected on the same day with the same dose of *S. mansoni* cercariae. On day 45 post infection all 16 mice were treated with a single oral dose of 120 µg praziquantel per gram body weight. This dose was known to eliminate *Schistosoma* in our model (own unpublished data). Groups of four mice were sacrificed on days 50, 80, 120, and 180 after infection, respectively, and from each group one mL of pooled plasma was tested. Figure 1 summarizes the CFPD target gene concentrations observed in all groups of mice, including the untreated group. Interestingly, in mice sacrificed five days after treatment the *Schistosoma* CFPD concentration in pooled plasma was considerably increased against the group that was sacrificed immediately before treatment (899.23 vs 128.27 target gene copies per mL). CFPD concentrations decreased to 182.93 and 70.97 target gene copies/mL on days 80 and 120, respectively, and became undetectable in the last group sampled on day 135 post treatment. It was concluded that the concentration of CFPD in plasma might be associated with the number of viable parasites or eggs in the mouse model, and the observed increase of CFPD immediately after treatment may have been due to parasite decay.

To determine whether CFPD could also be detected in humans, fourteen patients with chronic disease were studied. These patients had been referred to our tropical medicine ward after being identified in routine screening for gastrointestinal conditions or other symptoms compatible with Schistosomiasis. It could not be reconstructed how long these patients had been infected, or how long ago they had been exposed. Diagnoses were initially made by EIA. Active infections were subsequently confirmed in all patients by microscopic detection of intact eggs in urine, stool, or organ biopsies. Either *S. mansoni*, or *S. haematobium*, or *S. japonicum* eggs were seen (Table 1). From each patient, 10 mL of plasma were extracted and tested for *Schistosoma* CFPD. All patients tested positive. The observed CFPD concentrations ranged from 1.22 to 27,930 target gene copies per mL of plasma.

**Table 1:** Patients with chronic disease.

Patient	Suspected origin of infection	Country of residence	Residence status	EIA	*Schistosoma* species	Sample in which eggs were detected	Cell-free DNA cop/mL^a^
1	West Africa	No data available	Refugee	+	*S. haematobium*	Bladder biopsy	27930.64
2	Mozambique	Germany	NGO worker	*+*	*S. mansoni*	Rectum biopsy	27930.64
3	Nigeria	Nigeria	Immigrant	*+*	*S. mansoni*	Rectum biopsy	3247.14
4	Egypt	Egypt	Immigrant	*+*	*S. mansoni*	Rectum biopsy	1584.80
5	Egypt	Egypt	Immigrant	*+*	*S. mansoni*	Rectum biopsy	1584.80
6	Philippines	Germany	Expatriate	*+*	*S. japonicum*	Rectum biopsy	1584.80
7	Egypt	Egypt	Immigrant	*+*	*S. mansoni*	Rectum biopsy	773.48
8	Zambia	Germany	NGO worker	*+*	*S. mansoni*	Rectum biopsy	377.50
9	West Africa	No data available	Refugee	*+*	*S. mansoni*	Rectum biopsy	184.24
10	Ghana	Ghana	Immigrant	*+*	*S. mansoni*	Rectum biopsy	184.24
11	Gambia/Senegal	Gambia/Senegal	Immigrant	*+*	*S. mansoni*	Rectum biopsy	21.42
12	West Africa	West Africa	Immigrant	*+*	*S. haematobium*	Urine	21.42
13	Uganda	Uganda	Immigrant	*+*	*S. mansoni*	Stool	2.49
14	Egypt	Egypt	Immigrant	*+*	*S. mansoni*	Rectum biopsy	1.22

a Note that 10 mL of plasma were processed. 1 copy per mL = 1.67 copies per PCR vial.

**Figure 2:**
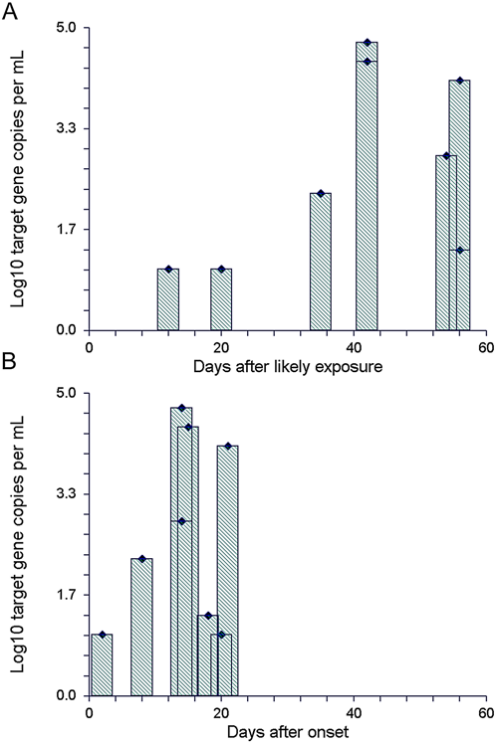
Cell-free *Schistosoma* DNA concentrations in plasma of patients with acute disease (Katayama syndrome) plotted against the days post exposure or post onset of symptoms when the tested samples were taken. 10 mL of plasma were tested for cell-free DNA.

Because of the high detection rates in patients with active disease, it was tested whether CFPD might already be detectable in the early acute disease (Katayama syndrome). Eight patients were studied, as shown in Table 2. All of these patients had acute disease that was confirmed subsequently to be associated with *Schistosoma* infection. Although most patients were seen only in the third week of symptoms, two patients could be tested already on days 2 and 8 of symptoms, respectively. In three of eight patients, antibody EIA was still negative during the first visit. CFPD PCR was positive in all eight patients (Table 2). Target gene concentrations in the cohort seemed to increase with increasing times after exposure or after disease onset, as shown in Figure 2. Highest values were observed about six weeks from exposure or about 15 days from onset of symptoms.

**Table 2:** Patients with Katayama syndrome.

Patient	Destination	Purpose	Visit	DPE^a^	DPO^b^	DPT^c^	LEUK^d^	EO^e^	EIA^f^	Cell-free DNA copies per mL^g^
1	Mozambique	Professional	First	42	14		13.6	20.9	+	57227.85
			Second	210	195	156	5.3	3.6	+	21.42
2	Ethiopia	Professional	First	42	15		10.2	26	−	27930.64
			Second	135	120	79	7.3	4.1	+	1584.80
3	Uganda	Professional	First	56	18		10.1	19	+	21.42
			Second	270	250	200	4.9	2.6	+	5.10
4	Uganda	Professional	First	12	20		7.3	22	+	10.45
			Second	460	445	434	6.9	5.0	+	2.49
5	Malawi	Tourist	First	20	2		6.2	6.5	+	10.45
			Second	750	740	716	5.2	0.8	+	−
6	Mozambique	Tourist	First	56	21		50.7	65	−	13631.84
7	Jemen	Tourist	First	54	14		6.7	23	+	773.48
8	Malawi	Tourist	First	35	8		4.9	19.7	−	184.24

a Days post exposure with fresh water (most likely event).

b Days post onset of symptoms.

c Days post treatment for second visits.

d Leukocyte count (n per nL). Average leukocyte count in patients 1 to 5: first visit, 9.48 cells/nl; second visit, 5.92 cells/nl (p<0.0017).

e Percent eosinophiles in total leukocytes. Average eosinophile fraction in patients 1 to 5: first visit, 18.88%; second visit: 3.2% (p<0.033).

f Enzyme immunoassay.

g Note that 10 mL of plasma were processed. 1 copy per mL = 1.67 copies per PCR vial.

It was next studied whether a decrease of CFPD concentration due to treatment could be confirmed. In the group of patients with Katayama syndrome, five of eight patients could be followed after treatment (Table 2). All five patients received praziquantel and prednisolon (1 mg/kg) within two weeks after initial diagnosis. A second treatment course (same dose of praziquantel, no prednisolon) was conducted in all patients 4 to 6 weeks later. Patients were appointed for control visits which took place 105 to 738 days after the initial visit (rows labelled “second visit” in Table 2). As expected, average leukocyte counts and levels of eosinophilia (% eosinophiles in leukocyte count) were significantly lower in second visits than in first visits. All patients had normal or only marginally increased eosinophile levels during their second visits (Table 2). Mean *Schistosoma* CFPD target gene concentrations in plasma were 17,040.20 copies/mL during first visits and 322.76 copies/mL during second visits. Means were significantly different (two-tailed T-test, p<0.05, Wilcoxon paired-sample test, p<0.04). Interestingly, only one patient had a completely negative CFPD PCR test during the second visit, and this was the patient with the longest interval between treatment and second visit.

To obtain more data on *Schistosoma* CFPD concentrations after treatment, we tested 30 patients who had been treated for schistosomiasis during eight years in our institution, and who were available for a re-visit. These patients were in good clinical condition, had no eosinophilia, and had received between 1 and 6 treatment courses since their last exposure in endemic regions. Patient histories are summarized in Table 3. Ten of the 30 patients had positive CFPD PCR results. Intervals between treatment and PCR testing were significantly different between PCR-positive and PCR-negative patients (0.43 years vs. 3.4 years, p<0.0004, ANOVA f-test). The longest interval between treatment and a positive PCR result in any patient was 58 weeks. Interestingly, three of the ten patients with positive PCR showed dead eggs in histology.

**Table 3:** Patients seen after treatment.

Patient	Country of origin	Residence status	*Schistosoma* species^a^	Histology^b^	YPE^c^	NT^d^	TPT^e^	Cell-free DNA cop/mL^f^
1	Egypt	Immigrant	*S. haematobium*	Negative	3	1	2 wk	6653.16
2	Sierra Leone	Refugee	Unclassified	Negative	6	1	2 wk	89.92
3	Guinea	Immigrant	*S. mansoni*	DE	8	1	4 wk	89.92
4	Sierra Leone	Refugee	Unclassified	Negative	7	2	13 wk	43.88
5	Zimbabwe/Botswana	Immigrant	Unclassified	Negative	4	1	2 wk	21.41
6	German	Tourist	Unclassified	Negative	3	3	24 wk	10.45
7	Ghana	Immigrant	*S. mansoni*	EO	2	4	54 wk	10.45
8	Data not available	Immigrant	Unclassified	DE	2	2	58 wk	2.49
9	Germany	Expatriate	Unclassified	DE	3	2	9 wk	2.49
10	Egypt	Immigrant	Unclassified	Negative	4	5	52 wk	2.49
11	Cameroon	Immigrant	*S. haematobium*	Negative	2	3	2 y	-
12	Uganda	Immigrant	Unclassified	Negative	3	1	0 y	-
13	Germany	Tourist	*S. haematobium*	Negative	3	3	1 y	-
14	Germany	Tourist	*S. mansoni*	Negative	3	3	2 y	-
15	Philippines	Immigrant	*S. japonicum*	DE	4	6	2 y	-
16	Ghana	Immigrant	Unclassified	EO	4	2	3 y	-
17	Germany	Tourist	*S. mansoni*	Negative	4	3	3 y	-
18	Egypt	Immigrant	*S. mansoni*	EO	5	1	0 y	-
19	Egypt	Immigrant	Unclassified	Negative	5	4	3 y	-
20	Cameroon	Immigrant	Unclassified	EO	6	2	5 y	-
21	Egypt	Immigrant	*S. mansoni*	Negative	6	4	4 y	-
22	Ghana	Immigrant	*S. haematobium*	DE/EO	10	1	0 y	-
23	Germany	Tourist	Unclassified	Negative	10	3	8 y	-
24	Egypt	Immigrant	*S. haematobium*	Negative	6	2	6 y	-
25	Germany	Expatriate	Unclassified	Negative	5	3	4 y	-
26	Cameroon	Immigrant	Data not available	Negative	5	3	4 y	-
27	Germany	Tourist	Unclassified	Negative	5	3	4 y	-
28	Cameroon	Immigrant	Unclassified	Negative	6	3	5 y	-
29	Egypt	Immigrant	Unclassified	Negative	7	4	4 y	-
30	Data not available	Immigrant	Unclassified	DE	8	2	8 y	-

a Identified by microscopy during earlier active disease episode (recorded data).

b Microscopic findings in colon or bladder biopsy upon re-visit. DE = degenerated eggs; EO = eosinophilic infiltrates; Negative = normal histology.

c Years post exposure = time (years) between last exposure in endemic country and PCR testing.

d Number of earlier treatment courses since last exposure.

e Time post treatment = time (wk = weeks; y = years) between completion of last treatment course and PCR testing.

f Note that 10 mL plasma were processed. 1 copy per PCR mL = 1.67 copies per PCR vial.

**Figure 3:**
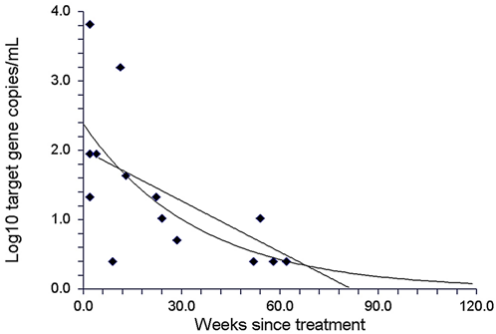
Cell-free *Schistosoma* DNA concentrations after treatment. DNA concentrations were plotted only for those patients still showing cell-free *Schistosoma* DNA in plasma after treatment. These data were pooled from patients who had been followed prospectively after being diagnosed with Katayama syndrome, as well as from patients examined retrospectively after concluded treatment. Linear regression analysis yielded the graph equation Y = 2.03−0.02 X. Exponential regression yielded the graph equation Y = e ^∧^ −0.02 (X−30.4).

To obtain an estimate of the approximate duration of CFPD detection after therapy, the CFPD target gene concentrations were plotted against time for all patients in this study who provided positive PCR results after treatment (patients from the Katayama syndrome ever group and post-treatment group). As shown in Figure 3, linear regression or exponential curve fitting suggested that negative results could be expected by weeks 82 or 120 after treatment, respectively.

## Discussion

Schistosomiasis involves a wide range of symptoms and is difficult to diagnose. In this study we have explored the utility of detecting cell-free parasite DNA (CFPD) in serum as an alternative to detecting eggs in stool, urine, or organ biopsies. The concept of using cell-free DNA for diagnostic purposes has been proven in oncology and prenatal diagnostics [25]–[33]. It was our rationale that schistosomiasis involves parasite turnover, liberating DNA from decaying parasites that would reach the blood. Unlike eggs in stool or urine, CFPD in plasma would not undergo random sampling effects that complicate diagnostics.

By means of a well-established murine model of schistosomiasis, it was confirmed that DNA could be detected in plasma during active disease, and that praziquantel treatment led to clearance of *Schistosoma* CFPD from plasma. Consistent with the hypothesis that circulating *Schistosoma* DNA stemmed from decaying parasites, a marked increase of CFPD concentration was observed in plasma of mice sampled short after initiation of therapy.

Because of the large differences in plasma volume between mice and humans, we have not undertaken any further mouse experimentation but continued a proof-of-concept study on available patients with schistosomiasis in various clinical stages. In a first approach, we showed that CFPD could be detected in all of 14 patients with active disease. Due to the small number of available patients, this finding clearly awaits confirmation in larger studies. It should also be mentioned that the sensitivity of our assay may vary between *Schistosoma* species, as the target gene has not been formally evaluated in *S. japonicum* (e.g., our whole study contained only one patient with *S. japonicum*), and it has been shown that *S. hematobium* contains less copies of it than *S. mansoni* [40]. More recent PCR protocols (e.g., [21]) may be better suited to detect all species with the same sensitivity. This study therefore does clearly not provide a protocol intended for direct transfer into clinical application. Nevertheless, it is an interesting perspective that CFPD PCR might reach a clinical sensitivity of 100% for active schistosomiasis. In industrialized countries, it may be easier to find well-equipped molecular diagnostic laboratories than experienced microscopists with sufficient expertise in *Schistosoma* egg detection. Because of the ease of taking blood samples, and in view of the risk contributed by undiagnosed Schistosomiasis, it could become a realistic option to integrate *Schistosoma* CFPD PCR in routine diagnostic regimens for the clarification of gastrointestinal or urological conditions.

Katayama syndrome caused by acute *Schistosoma* infection is a major differential diagnosis in returning travellers presenting with fever of unknown origin [6]. Although eosinophilia is a helpful criterion to distinguish Katayama syndrome from other conditions such as malaria or dengue fever, it is difficult to make a distinctive diagnosis due the shortcomings of serology and the inability of demonstrating *Schistosoma* infection before egg production [14]. We have demonstrated here that CFPD can be detected very early after onset of symptoms in patients with Katayama syndrome. Despite the limited number of patients studied, the concentrations of CFPD observed in our patients were well above the detection limit of the PCR assay. Based on experiments on limiting dilution series and quantitative correction factors as described in the Materials and Methods section, it could be assumed that the technical sensitivity limit of our assay was ca. 1.67 CFPD target gene copies per mL of plasma. The earliest patient with Katayama syndrome sampled on day 2 of symptoms already had a plasma concentration of ca. 10 copies per mL. If larger studies can confirm the high clinical sensitivity seen in our study, the detection of CFPD in plasma might become an accepted way of ruling out Katayama syndrome. It should be mentioned here that we have meanwhile modified our protocol by testing smaller volumes of plasma (in the order of 1–2 mL) and using a larger input volume of DNA in PCR. This modification makes the method easier to handle in routine laboratories, and still seems to provide sufficient sensitivity to diagnose patients with Katayama syndrome.

**Figure 4:**
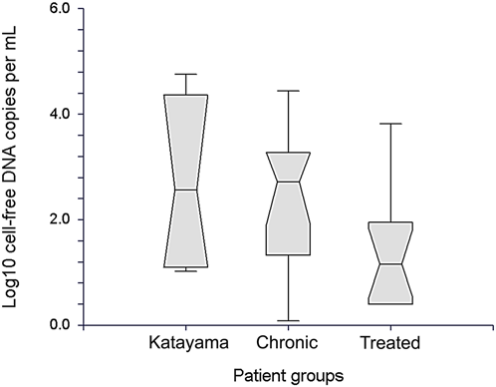
Box plot analysis of cell-free DNA concentrations in patients with Katayama syndrome (first visits only), patients with chronic disease, and all patients who had positive plasma PCR after treatment (pooled from treated Katayama syndrome patients and patients examined retrospectively after treatment). Boxes represent the innermost two quartiles (25%–75% percentiles = interquartile range, IQR) of data. The whiskers represent an extension of the 25^th^ or 75^th^ percentiles by 1.5 times the IQR. The notches represent the median +/−1.57 IQR √n. If the notches of two boxes do not overlap, the medians ( = notch centers) are significantly different (true for the active disease vs. the treated group).

A third field of application is the monitoring of therapy. In order to prevent relapse and long term sequelae from insufficient treatment, it is important to achieve a laboratory confirmation of the success of treatment [18], [43]–[45]. Unfortunately, patients after therapy as well as patients after a long course of disease with spontaneous healing (“burnt out bilharzia”) are difficult to judge based on clinical or laboratory findings [16],[18]. Several repetitive, parallel samplings are necessary to increase the statistical chance of detection of eggs by microscope, and thus to increase the clinical sensitivity of laboratory diagnostics [20],[22],[46]. This problem applies not only to microscopy, but also to conventional PCR on stool or urine samples [21],[47]. In the latter tests, there are additional issues such as PCR inhibition in stool samples. We have shown here that the concentration of CFPD in plasma was significantly reduced after therapy. The average CFPD concentration in those patients who still had detectable DNA after treatment (25.1 copies per mL) was significantly lower than in patients with Katayama syndrome (first visits, 537 copies per mL) or active disease (323.6 copies per mL), as determined by ANOVA (F-test, p<0.035; refer to Figure 4 for a Box Plot diagram). The decline of CFPD concentration in patients before and after treatment may thus become an effective parameter for monitoring patients under therapy. On the contrary, we were surprised to see that it took considerably longer in humans than in mice for CFPD PCR to become entirely negative after treatment. Lo et al. have determined that the half-life of fetal DNA in mother's plasma after birth ranges between 4 and 30 minutes [48]. In our study, pooled data from patients followed prospectively and patients re-examined retrospectively after treatment suggest that it may take more than one year until CFPD becomes entirely undetectable. Although we have no experimental evidence, it can be speculated that inactive eggs may release DNA with very slow kinetics. The greater number of eggs in humans with chronic disease as opposed to mice in our experiments may be responsible for a considerably longer duration until CFPD is totally eliminated in humans. Future studies should address the utility of paired CFPD determinations in individual patients before and after treatment, rather than insisting on negative CFPD results for a confirmation of treatment success.

In summary, the detection and quantification of CFPD from plasma might carry the potential of becoming a novel diagnostic tool for any stage of schistosomiasis. With increased automation and better instrumentation for molecular diagnostics, the cost efficiency and quality of results in clinical laboratories can exceed that of repetitive diagnostic determinations by microscopy. The cost of reagents and consumables for our method range around 3 USD per determination, which is probably too expensive in many endemic countries. However, this price is compatible with application in funded surveillance and control programmes, and should be affordable for individualized application in emerging countries. Instrumentation and expertise for proper PCR diagnostics has considerably improved in many countries due to the demands created by HIV and TBC treatment programmes. If future studies can prove the clinical benefits suggested here, *Schistosoma* CFPD PCR may become a new priority in molecular diagnostics in developing and emerging countries.
